# Surgical Management of Left Atrial Myxoma through Right Mini-Thoracotomy in a Patient with Retrosternal Gastric Tube

**DOI:** 10.70352/scrj.cr.26-0293

**Published:** 2026-06-26

**Authors:** Masayuki Nishiyama, Takayuki Okada, Tadaaki Koyama

**Affiliations:** Department of Cardiovascular Surgery, Kansai Medical University, Hirakata, Osaka, Japan

**Keywords:** left atrial myxoma, retrosternal gastric tube, right mini-thoracotomy, minimally invasive cardiac surgery

## Abstract

**INTRODUCTION:**

Open-heart surgery in patients with a retrosternal gastric tube after esophagectomy presents a significant surgical challenge owing to the risk of conduit injury during median sternotomy. In such cases, a minimally invasive cardiac surgery (MICS) strategy to avoid sternotomy is of paramount importance and must be tailored according to individual anatomy.

**CASE PRESENTATION:**

A 71-year-old man with a history of robot-assisted subtotal esophagectomy and retrosternal gastric tube reconstruction was found to have a mobile left atrial mass attached to the interatrial septum. Preoperative CT showed that the gastric conduit was located immediately beneath the sternum, indicating a high risk of injury during median sternotomy. Therefore, a MICS approach via right mini-thoracotomy was selected to avoid sternotomy. The procedure was performed through a right inframammary incision via the fourth intercostal space. Cardiopulmonary bypass was established via femoral arterial cannulation and direct bicaval venous cannulation. The tumor was successfully resected through a transseptal approach, and the atrial septum was reconstructed using an autologous pericardial patch. A transesophageal echocardiography probe was inserted carefully without resistance or complications, even despite the presence of a reconstructed gastric conduit. Intraoperative findings confirmed that the gastric tube was intact. The postoperative course was uneventful. Histopathological examination confirmed the diagnosis of cardiac myxoma and its complete resection.

**CONCLUSIONS:**

A sternotomy-avoiding MICS strategy via right mini-thoracotomy is a safe and effective approach for left atrial tumor resection in patients with a retrosternal gastric tube. Careful preoperative imaging and strategic surgical planning are essential to prevent catastrophic complications and optimize outcomes.

## Abbreviation

MICS
: minimally invasive cardiac surgery

## INTRODUCTION

Open-heart surgery in patients with a retrosternal reconstructed gastric conduit following esophagectomy presents a significant surgical challenge. Median sternotomy, the standard approach for cardiac surgery, is associated with a substantial risk of injury to the reconstructed gastric tube, which may lead to catastrophic complications such as mediastinitis or the need for complex re-reconstruction. MICS strategies, such as right mini-thoracotomy, have emerged as alternative strategies to avoid these risks. However, prior thoracic surgery may result in pleural adhesions, making such approaches technically demanding.

Herein, we report a case of left atrial myxoma successfully resected via right mini-thoracotomy in a patient with a history of robot-assisted esophagectomy and retrosternal gastric tube reconstruction. To the best of our knowledge, very few reports have described right mini-thoracotomy for left atrial tumor resection after robot-assisted esophagectomy with retrosternal gastric conduit reconstruction are extremely limited.

## CASE PRESENTATION

**Fig. 1:**
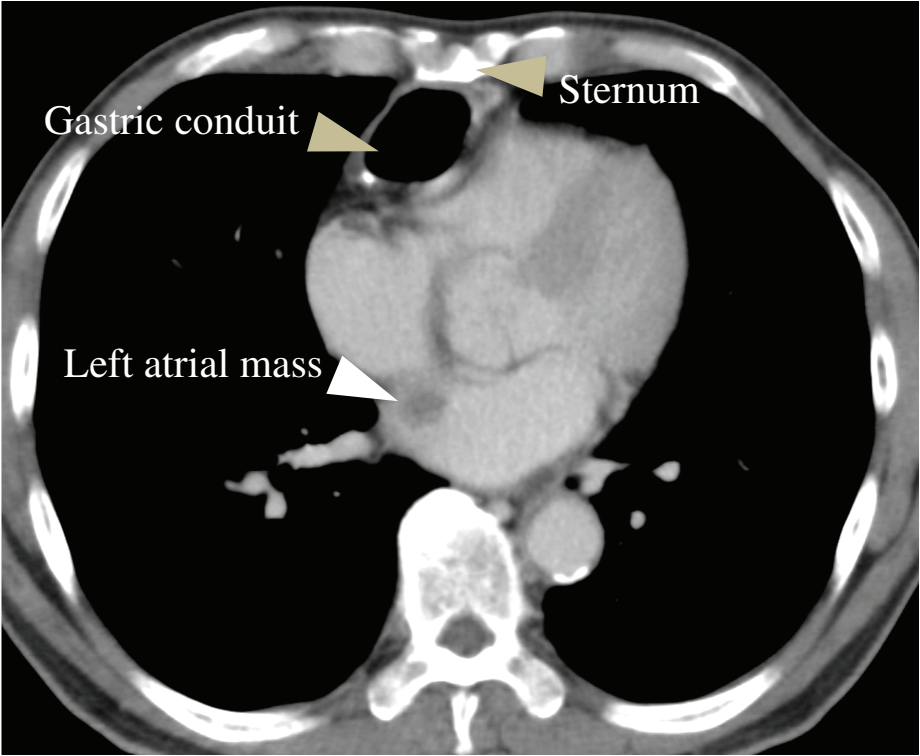
Contrast-enhanced CT showing a retrosternal reconstructed gastric conduit adjacent to the sternum (gray arrows) and a sessile left atrial mass (white arrow).

**Fig. 2:**
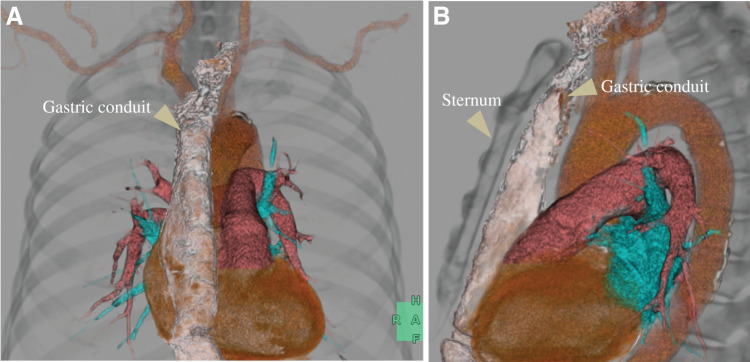
Bone-removed 3D CT demonstrating the gastric conduit behind the sternum (gray arrows). (**A**) Anterior view. (**B**) Left lateral view.

A 71-year-old man with a history of esophageal cancer underwent robot-assisted subtotal esophagectomy, followed by retrosternal gastric tube reconstruction 8 months prior to presentation. Postoperative CT and transthoracic echocardiography revealed a mobile left atrial mass attached to the interatrial septum. Thus, surgical resection was indicated. Preoperative CT showed that the reconstructed gastric tube immediately beneath the sternum, suggesting the presence of dense adhesions between the gastric conduit and the posterior surface of the sternum (**Figs. 1** and **2**). Therefore, median sternotomy was considered high risk for gastric tube injury and was avoided. A right mini-thoracotomy approach was selected. A transesophageal echocardiography probe was carefully inserted through the reconstructed gastric conduit without resistance or complications. Although transesophageal echocardiography after esophagectomy is generally considered challenging because of altered anatomy, careful insertion under close monitoring allowed safe use in this case. Nevertheless, the potential risk of conduit injury should be recognized. The patient was assisted into the left semi-lateral decubitus position. An 11-cm right inframammary transverse incision was made, and the chest was entered through the fourth intercostal space.

### Surgical technique

**Fig. 3:**
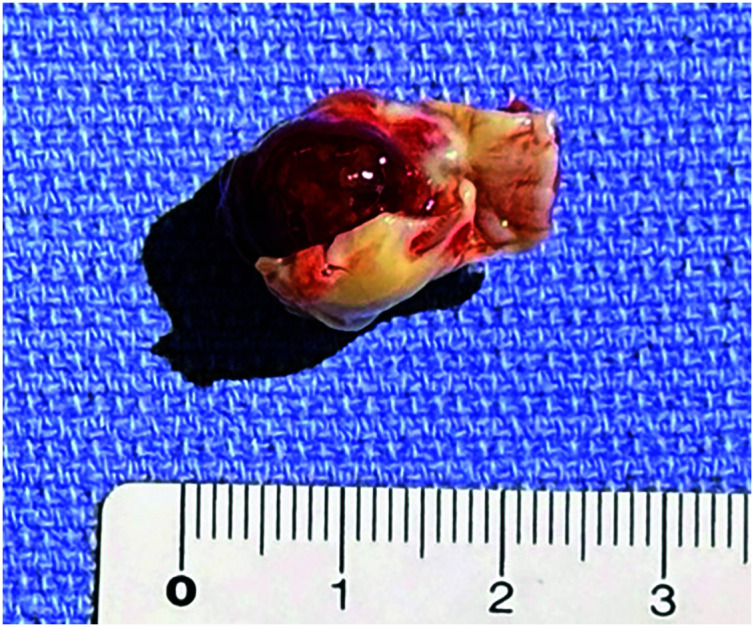
Resected tumor specimen (20 mm).

**Fig. 4:**
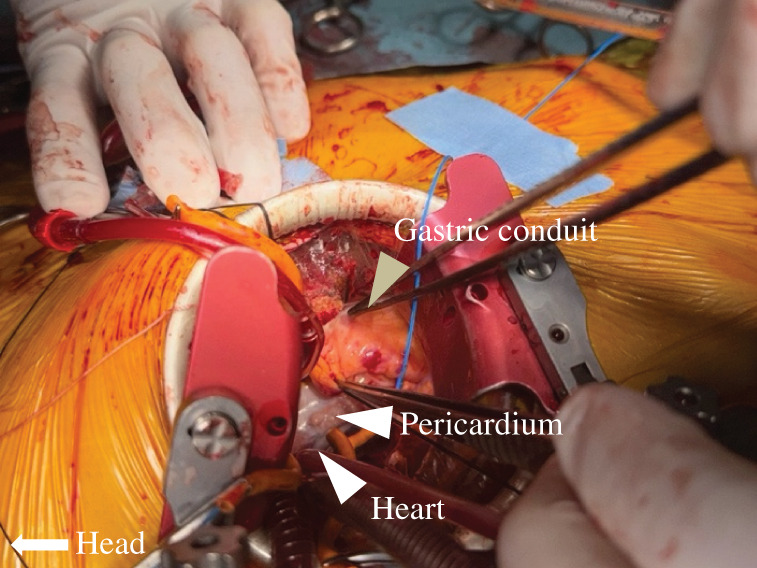
Intraoperative view showing the gastric conduit (gray arrow). The heart and pericardium are indicated by white arrows.

Intraoperatively, minimal pleuropulmonary adhesions were observed. The pericardium was opened anterior to the right phrenic nerve, ensuring that the dissection remained lateral to the presumed location of the gastric conduit. Preoperative imaging findings were referenced intraoperatively to avoid dissection toward the substernal space where the conduit was located. The reconstructed gastric tube was not directly manipulated. From the operative field, the gastric conduit was visualized on the anterior aspect of the heart beyond the pericardium, confirming its close proximity while remaining safely separated from the surgical field. Arterial cannulation was established via the right femoral artery. Attempts at bilateral femoral venous cannulation were unsuccessful; therefore, pericardiotomy was performed, followed by direct bicaval cannulation of the superior and inferior vena cava to establish cardiopulmonary bypass. The ascending aorta was cross-clamped using a direct transthoracic clamp through the thoracotomy incision. Myocardial protection was achieved with antegrade cold blood cardioplegia delivered via the aortic root, with repeated doses administered as needed. The procedure was performed under direct vision without endoscopic assistance, and pericardial traction sutures enabled adequate exposure and optimized patient positioning. The left atrium was accessed through a transseptal approach. The tumor, attached to the interatrial septum, was completely excised (**Fig. 3**). The resulting atrial septal defect was reconstructed using an autologous pericardial patch. From the operative field, the reconstructed gastric tube was clearly visualized on the anterior surface of the heart, and no injury was observed (**Fig. 4**). Histopathological examination confirmed complete resection with negative margins and established the diagnosis of cardiac myxoma. The postoperative course was uneventful, with no evidence of gastric tube-related complications.

## DISCUSSION

Cardiac surgery in patients with a retrosternal gastric conduit after esophagectomy is rarely performed and technically challenging.^1)^ The major concern with median sternotomy is the risk of gastric tube injury, which can result in severe complications, such as mediastinitis, conduit loss, and the need for re-reconstruction using colonic interposition. Although some reports have described safe dissection between the sternum and gastric conduit, the risk remains substantial, particularly when preoperative imaging suggests dense adhesions.^2,3)^ In the present case, CT clearly demonstrated close proximity between the sternum and gastric tube, supporting the decision to avoid sternotomy. Right mini-thoracotomy provides a valuable alternative, offering direct access to the left atrium while avoiding the substernal space.^4,5)^ Compared with conventional full thoracotomy, this minimally invasive approach reduces surgical trauma while maintaining adequate exposure. Right mini-thoracotomy offers several potential advantages in this setting. Compared with conventional full thoracotomy, this minimally invasive approach may reduce chest wall trauma, postoperative pain, and respiratory impairment while promoting earlier postoperative recovery. In patients with a retrosternal gastric conduit, avoidance of substernal dissection may substantially reduce the risk of conduit injury. To our knowledge, this is the first reported case of cardiac surgery via right mini-thoracotomy following robot-assisted esophagectomy with retrosternal gastric conduit reconstruction. Therefore, this case may provide additional insight into the feasibility of a sternotomy-avoiding MICS strategy in this anatomically challenging case. Preoperative CT is pivotal in surgical planning. Detailed anatomical assessment allowed precise identification of the reconstructed gastric conduit and facilitated estimation of the risk of substernal adhesions. Furthermore, CT findings helped determine the optimal pericardial incision site and supported safe operative planning. Pleural adhesions represent another important concern after prior thoracic surgery. In the present case, adhesions were minimal and did not interfere with exposure. However, in patients with severe pleural adhesions, adhesiolysis may be technically challenging, and early initiation of cardiopulmonary bypass could facilitate safer management. Another consideration is the feasibility of transesophageal echocardiography after esophageal reconstruction. Although insertion through a reconstructed gastric conduit is often challenging because of altered anatomy, careful probe insertion under close monitoring allowed safe use without complications in the present case.

## CONCLUSIONS

Right mini-thoracotomy as part of a sternotomy-avoiding MICS strategy appears feasible and useful for left atrial tumor resection in patients with a retrosternal gastric tube. This approach enables feasible tumor resection while minimizing the risk of conduit injury associated with median sternotomy. Careful preoperative imaging assessment and meticulous surgical planning are essential to maximize the benefits of this strategy and optimize clinical outcomes.
